# Image-guided chemistry altering biology: An *in vivo* study of thermoembolization

**DOI:** 10.1371/journal.pone.0200471

**Published:** 2018-07-16

**Authors:** Erik N. K. Cressman, Chunxiao Guo, Niloofar Karbasian

**Affiliations:** Department of Interventional Radiology, University of Texas MD Anderson Cancer Center, Houston, Texas, United States of America; Virginia Commonwealth University, UNITED STATES

## Abstract

**Rationale:**

Advances in image-guided drug delivery for liver cancer have shown a significant survival benefit. However, incomplete treatment is common and residual disease is often found in explanted liver specimens. In addition, the need to treat a malignancy from multiple mechanisms at the same time for optimal outcomes is becoming more widely appreciated. To address this, we hypothesized that an exothermic chemical reaction could be performed in situ. Such a strategy could in principle combine several angles of attack, including ischemia, hyperthermia, acidic protein denaturation, and metabolic modulation of the local environment.

**Methods:**

The University of Texas MD Anderson Cancer Center Institutional Animal Care and Use Committee approved this study. Outbred swine (25–35 kg, 5 control and 5 experimental) were treated under general anesthesia. Embolization was performed with coaxial microcatheter technique in a segmental hepatic arterial branch using either ethiodized oil as control or with thermoembolic solutionBlood samples were obtained before, immediately after, and the day following the procedure just before CT scans and euthanasia. Livers were explanted and samples were obtained for histologic analysis.

**Results:**

All animals survived the procedure and laboratory values of the control and experimental groups remained within normal limits. The control group had a diffuse or cloudy pattern of attenuation on follow-up CT scan the day after, consistent with gradual antegrade sinusoidal transit of the embolic material. The experimental group had clearly defined vascular casts with some degree of peripheral involvement. At histology, the control group samples had the appearance of normal liver, whereas the experimental group had coagulative necrosis in small pale, punctate areas extending several hundred microns away from the treated vessels and a brisk inflammatory response just outside the margins.

**Conclusion:**

In situ chemistry via thermoembolization shows early promise as a fundamentally new tactic for image-guided therapy of solid tumors.

## Introduction

As cancer treatments advance, mortality rates have generally decreased. Hepatocellular Carcinoma (HCC), however, stands out for several reasons. It is a leading cause of cancer death, with new diagnoses exceeding 750,000/yr and mortality nearly as high [1, 2]. Furthermore, whereas death rates are decreasing for many cancers, HCC is the fastest rising cause of cancer death in the United States [3]. Given that morbid obesity is increasing worldwide and that this is strongly associated with the incidence of fatty liver, inflammation, cirrhosis, and thus HCC, the problem is expected to become significantly worse in the coming years [4, 5].

Conventional chemotherapy has not had a meaningful impact on HCC [6]. It is only in the last decade, with the advent of targeted therapies such as sorafenib, that even a modest survival benefit has been demonstrated [7]. Unfortunately, the number of patients who tolerate the highest dose, which is the only one shown to provide a survival benefit, is low [8]. Newer targeted therapies such as regorafenib may confer a similar benefit in the range of 3 months, but adverse effects were reported in 100% of patients in a recent report [9]. While surgical resection of tumor and liver transplant can offer a significant survival benefit and even a cure in some cases, few patients qualify for either and the availability of donor organs is drastically overshadowed by the need. Complicating matters further, sorafenib has not shown a survival benefit as an adjuvant therapy after resection or ablation [10].

Stratifying the remainder (in fact the majority) of patients who are in need of alternate therapies is a significant challenge because HCC occurs most commonly in the setting of underlying cirrhotic liver disease. Any therapy that treats tumor must take into consideration the degree of compromise in liver function and how toxic such therapy is to the liver. While there are a number of algorithms [11, 12], the most commonly used is the Barcelona Clinic Liver Cancer [13] or BCLC system. It is one of the most useful clinically relevant systems, but even this does not factor in the molecular profile for a particular patient, how to address a single large tumor, does not address prognosis after treatment, nor how to address treatment failures [14].

Depending on the circumstances, the two most widely used methods for unresectable HCC patients are ablation and some variant of transarterial chemoembolization (TACE). Ablation uses either microwave [15, 16] (MW) or radiofrequency energy [17, 18] (RF) to heat tissues to temperatures that cause thermal coagulation. Embolization methods rely on placement of a catheter under image guidance to deliver therapy just upstream from a tumor via the arterial supply [19]. Thermal and embolic methods both have shortcomings, however. While good results are obtained with smaller tumors, tumor recurrence and incomplete treatment are far too common [20, 21].

Doing both procedures for the same tumor to address this issue has seen a resurgence although the concept at least two decades old [22]. This increases both risk and cost in all cases but with very modest incremental survival in some cases [23]. Adjuvant therapy after surgical resection or ablation with sorafenib has shown no additional survival benefit [10]. Taken together, these observations motivated us to investigate a new image-guided strategy that would simultaneously combine multiple methods to destroy tumor using minimally invasive techniques in a single procedure. This would not leave any time for cells to adapt and protect themselves against a subsequent stress, nor for collateral vessels to restore some level of perfusion prior to the second procedure.

**Fig 1:**
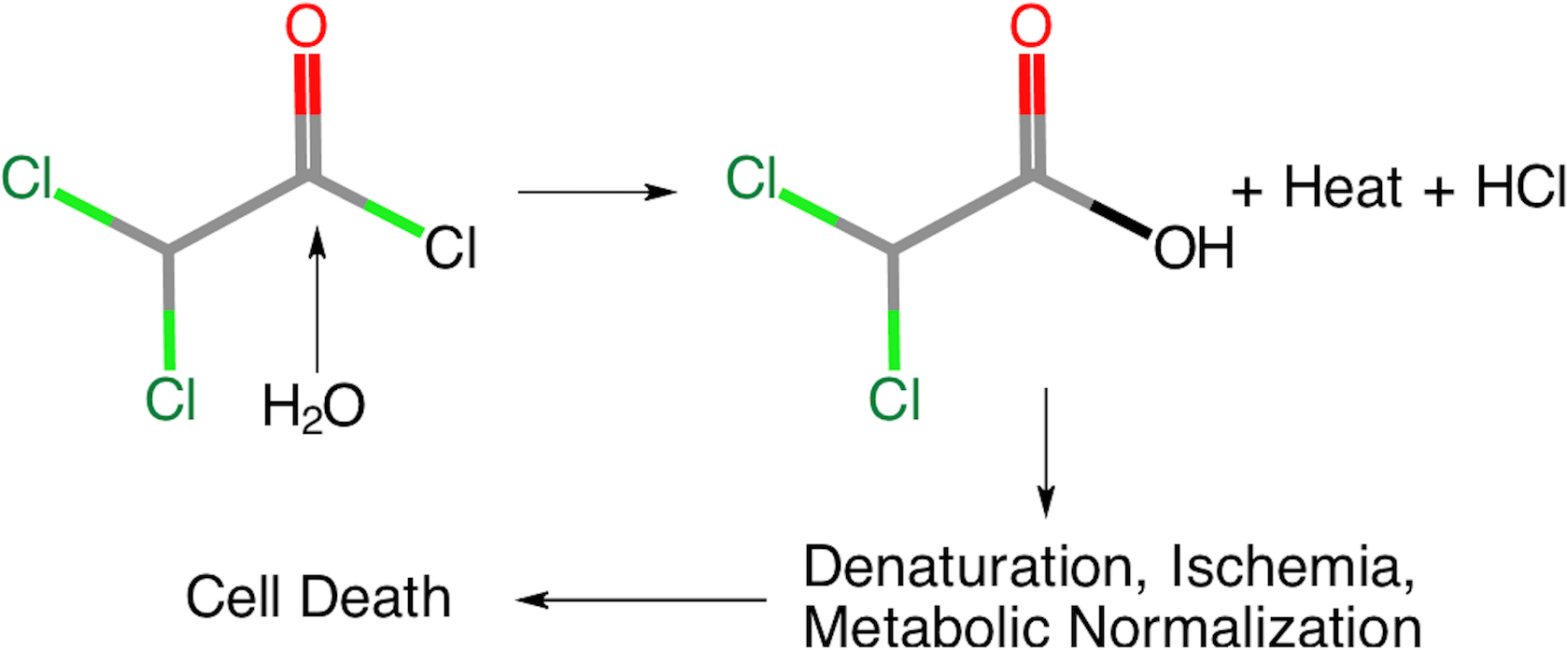
Illustration of electrophilic hydrolysis with water as the nucleophile and sequence of events thereafter. The water attacks and ultimately displaces the chloride ion on the carbonyl carbon, releasing -93 kJ/Mol of thermal energy [38]. At the same time, it generates HCl and the carboxylic acid. The two chlorine atoms on dichloroacetic acid lower the pKa to 1.26 making dichloroacetic acid also a strong acid. After protonation of a substrate occurs, the dichloroacetate anion remains and has intrinsic pharmacologic activity. Thus, heat, acid, drug, and ischemia are all combined in a single stroke, overwhelming cells and resulting in localized cell death.

An appealing strategy would be to use an exothermic reaction that would be targeted and delivered via the transcatheter route. We envisioned that such an exothermic reaction would occur with the vessel *itself* as well as within the vessel based on electrophilic hydrolysis (Fig 1). Exothermic chemistry[24–32] and heated embolic solutions [33–37] have both been reported previously, but endovascular chemistry is a new area of study. We chose dichloroacetyl chloride as the reagent for our investigation for two additional reasons. First, once hydrolyzed, the resulting carboxylic acid is also itself a strong acid that would contribute to the chemical ablative effect in addition to the exotherm and the ischemia. Second, the compound is also a drug that blocks aerobic glycolysis and shunts cells toward oxidative phosphorylation. In this report, we describe the acute in vivo effects of this procedure in a pig model. We examined local and systemic toxicity, and compared it to ethiodized oil as a control.

## Materials and methods

### General

This study was conducted with approval and oversight by the Institutional Animal Care and Use Committee (Study #00001478-RN00). Outbred swine (n = 10, 5 each in control and experimental groups) with weights ranging from 25–35 kg were used after several days of acclimation per institutional policy. Animals had free access to food and water and were fed a commercially available diet. Anesthesia was accomplished as previously described with minor alterations [39]. In brief, induction was accomplished by intramuscular Telazol (4.4 mg/kg) injection with maintenance using isoflurane (2.0%) and oxygen (0.8–1.5 liters/min). Buprenorphine (0.02mg/kg) was administered postoperatively as needed to provide analgesia per monitoring by the institution's veterinary medicine service. Blood draws were performed immediately prior to the embolization, immediately after completion of the procedure, and the following day just before sacrifice.

### Reagents

The acid chloride (DCA chloride, 98%, Sigma Aldrich, St. Louis, MO) was used directly as supplied. Ethiodized oil (Lipiodol, Guerbet, Princeton, NJ) was used as supplied. The acid chloride was dissolved in the ethiodized oil at a concentration of 2 mol/L using fresh solutions prepared just prior to each procedure.

### Experimental procedure

Using standard Seldinger technique under ultrasound guidance, access was obtained via common femoral artery with the animals in supine position. A 5F sheath was placed (COOK Medical, Bloomington, IN) and through this access a 5F Sos-2 catheter was negotiated into the origin of the celiac artery. Digital subtraction angiography from this location disclosed the target anatomy and a 2F coaxial microcatheter was then navigated to select segmental arteries for treatment. Embolization in the control group was performed by slow infusion of ethiodized oil (1.0–1.2 mL) with intermittent fluoroscopic monitoring to evaluate progress. Embolization in the experimental group was performed with a modified technique. Lidocaine (1%, 5 mL) was administered slowly over 1 minute prior to embolization. The thermoembolic solution (400 μL of 2 mol/L) was drawn up trapped between small aliquots of ethiodized oil (150 μL) at leading and trailing edges (total volume 700 μL) and delivered over approximately 1 minute. Hemostasis was achieved with manual compression after removal of all catheters and sheaths. The following day, animals were scanned and euthanized as described in detail below.

### Imaging

Fluoroscopic images for angiography were acquired using a portable C-arm unit. Computed tomography data were acquired on a 4-slice Lightspeed Plus scanner (General Electric Medical Systems, Milwaukee, WI, USA) at 120 kVp with a slice thickness of 5 mm. DICOM images were then post-processed using Osirix MD 8.5.1 (Pixmeo, Geneva, Switzerland). Reformats were produced in various projections with sculpting of extraneous tissue performed to disclose the liver. This was then windowed and leveled using a pseudo-color scheme to depict the distribution of embolic material in the liver.

### Necropsy

After CT scanning, animals were euthanized by overdose of Beuthanasia administered intravenously. The liver was removed intact and scanned with fiducial markers present to assist with sampling. Photographs of select sections were obtained and small portions then placed in formalin for further processing and staining.

### Statistical analysis

Mean values for each group were calculated at three different time points: before the experiment (Pre), immediately after (Post) and 24 hours after the experiment (24 Hour Post). In order to compare the serial laboratory profiles inside and between each group, paired and unpaired t-tests were used respectively. These values were calculated using Microsoft Excel. Values are expressed as mean ± standard deviation. P-values <0.05 were considered statistically significant.

## Results

### Angiography

**Fig 2:**
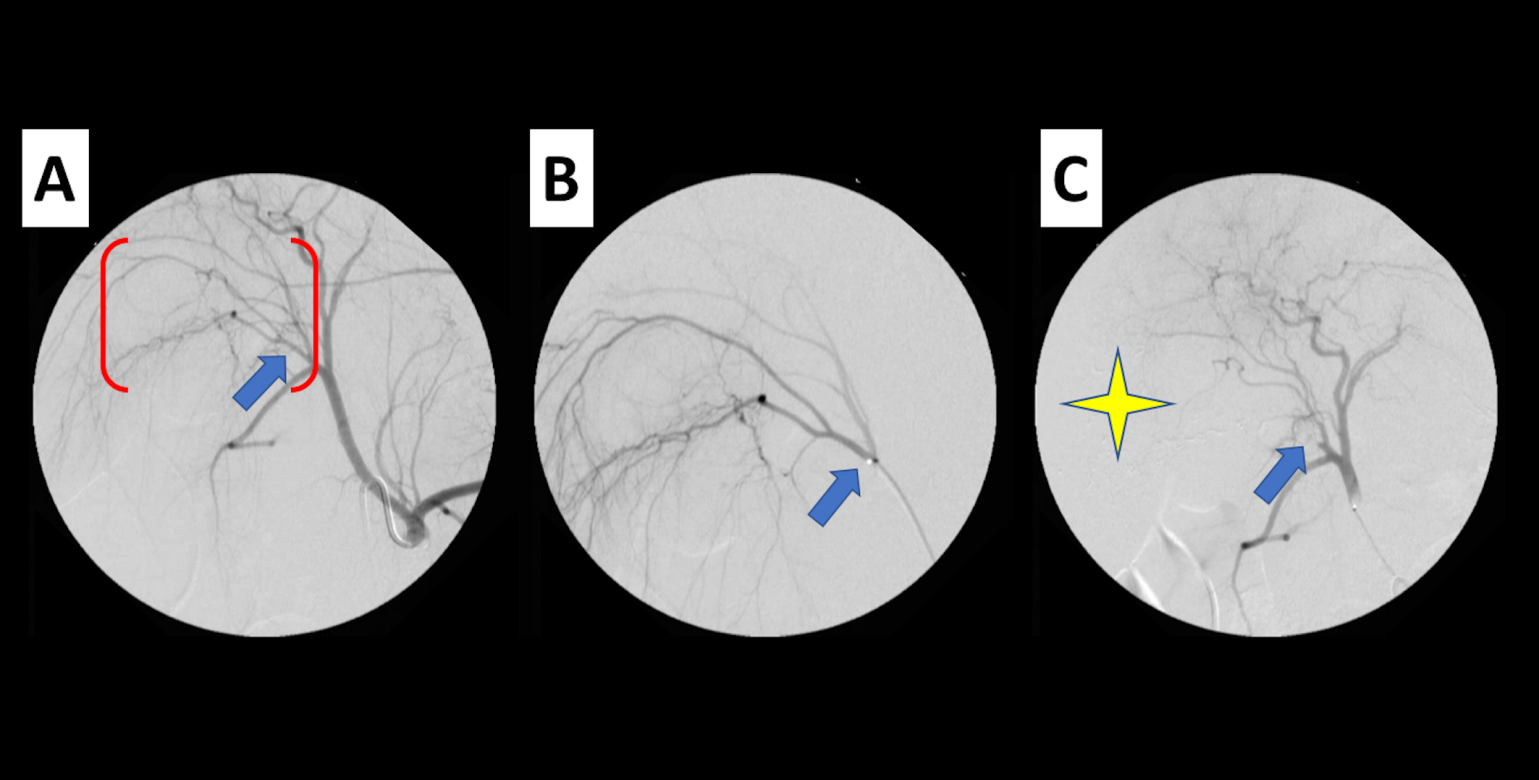
Image-guided targeting and delivery via thermoembolization. Representative case with effects shown by digital subtraction angiography of liver pre-, intra-, and post-embolization. (A) celiac anatomy with common hepatic artery and branches thereof. Blue arrow demonstrates right lateral hepatic artery target for segmental embolization and red brackets denote target vascular bed enlarged in next image (B) Enlarged view of target area (red bracket in A) with selective segmental angiography via microcatheter (tip indicated by blue arrow). Thermoembolic solution was delivered from this location. (C) Post-embolization, angiography clearly demonstrates remnant of target vessel and nonperfused area appreciated as absence of contrast during angiogram (star) when compared to image A.

In control animals using ethiodized oil, embolization was uneventful. The material was successfully delivered and over time the material moved progressively more distal in the vessels out to the capillary level. The thermoembolic solution was observed to flow distally in a similar manner but once stasis was reached in the segmental artery, the solution did not mover further distally. In the experimental group, stasis was observed in a very short time interval. The appearance did not change when angiography was repeated after several minutes to allow material to transit more distally (Fig 2).

### CT images

**Fig 3:**
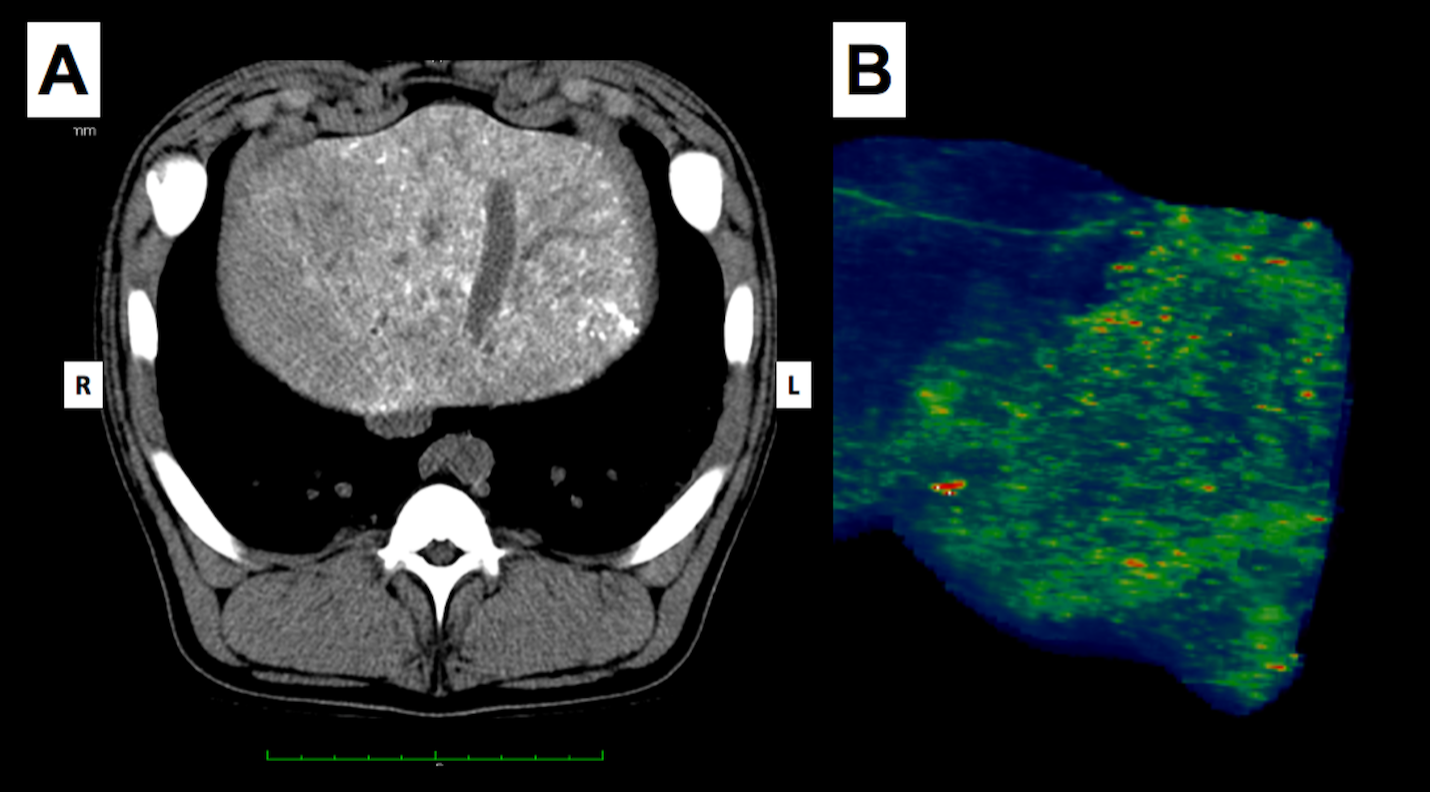
Control arm using vehicle (ethiodized oil) alone. Non-contrast CT image obtained 24 h post embolization (A) in situ and (B) reformatted showing distribution of embolic agent. Note contrast with experimental agent, with diffuse, cloud-like distal distribution pattern of liquid embolic and absence of linear vascular structures. This corresponds to movement out to the sinusoidal level. The pattern indicates that stasis did not occur in this arm of the study and thus minimal or no necrosis would be expected. This was confirmed at histology. In clinical practice, this pattern disappears over 3–4 weeks and is not apparent on follow-up scans.

**Fig 4:**
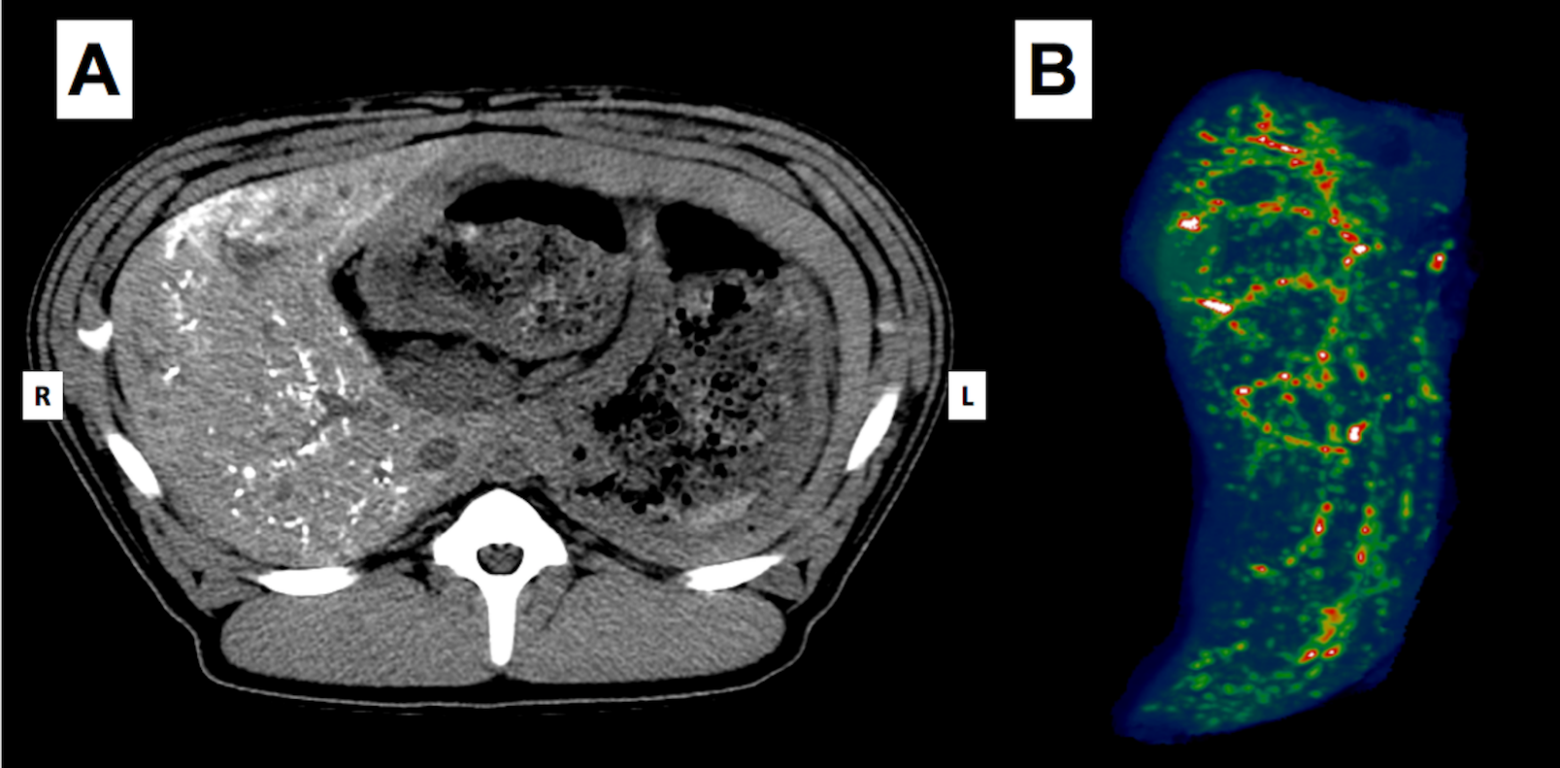
Experimental thermoembolization arm using 400 μL payload. Non-contrast CT image obtained 24 h post embolization (A) axial image in situ prior to euthanasia with small white areas on the right side within the liver, depicting thermoembolic solution in segmental and subsegmental branches of the right hepatic artery and (B) post-processed pseudo color, multi-planar volume reformat image. Note tree-like appearance of embolization due to the arterial route of administration; in addition, some indistinct areas in distal branches indicate penetration further to the arteriolar and sinusoidal levels. The persistence of the material in the vascular tree a full day after the procedure implies reaction occurred and that stasis was achieved, a finding which is corroborated at histology.

The day after the procedure, CT scans without contrast were obtained to map distribution of the embolic agent in each case. In the ethiodized oil group, the appearance was quite consistently more diffuse in character, with very few vessels discretely identified (Fig 3). This contrasted sharply with the appearance of the experimental group. In all animals in this group, the attenuation closely followed the course of the segmental and subsegmental vessels (Fig 4). Only a small portion in each case took on the cloudy appearance so characteristic of the more distally distributed ethiodized oil.

### Gross and histopathology

**Fig 5:**
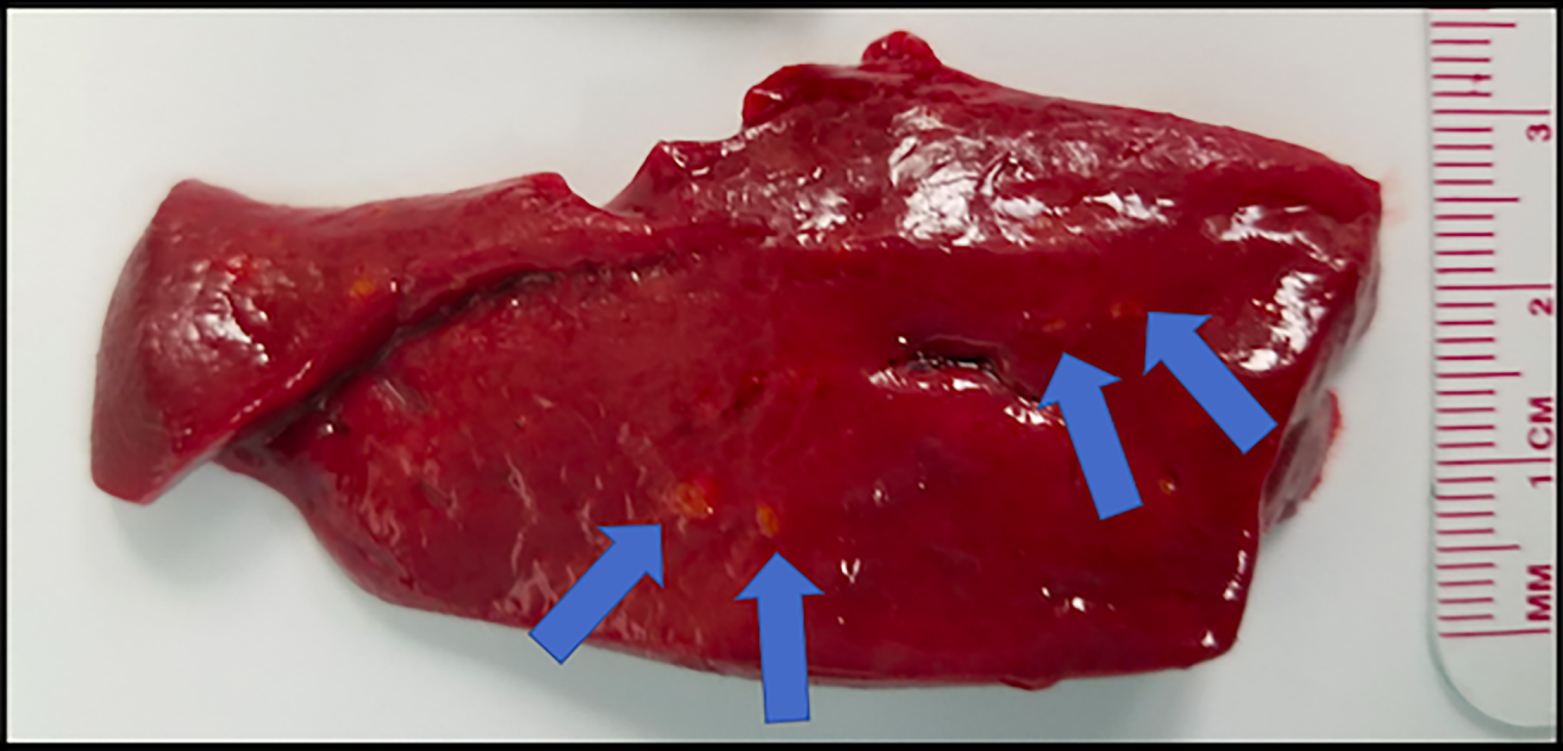
Gross pathology of section of treated lobe of liver. At this early stage (24 h after treatment) treated areas appear as small areas of light red to orange color (blue arrows) against the background of red parenchyma.

**Fig 6:**
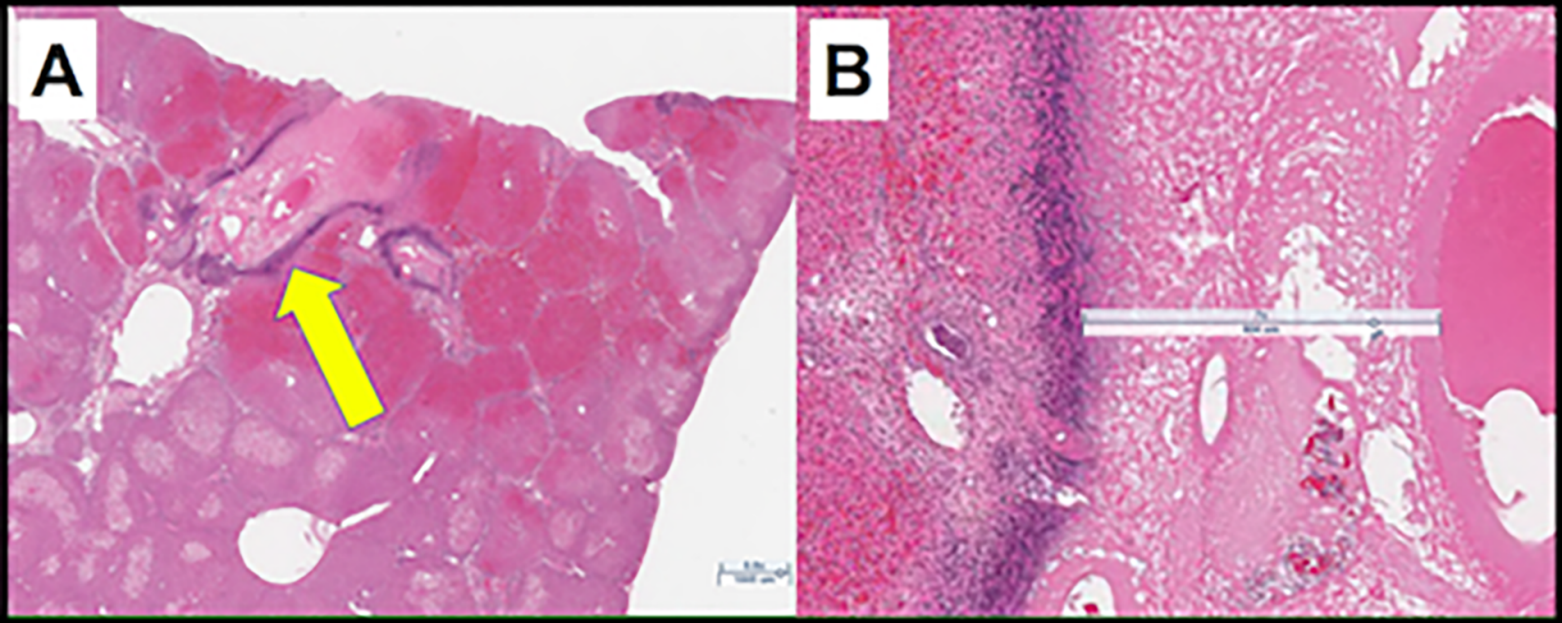
Histology of thermoembolization at 24 hours post-procedure with low and high power views. (A) Perivascular distribution of coagulative necrosis is clearly demonstrated with dense inflammatory infiltrate at margins (yellow arrow). (B) Magnification view of lesion in A: note the distance the necrosis extends from the vessel lumen and the amount of necrosis, particularly in view of the small dose employed (400 μL). Localized lymphocytic infiltrate and hemorrhage are present surrounding the lesions. Bar = 1000 μm (L) and 600 μm (R).

Examination of the freshly explanted liver in most cases revealed subtle changes at best, generally unremarkable. Small punctate pale areas could occasionally be identified, which were more easily appreciated on cut sections (Fig 5). Histologically, however, large areas of tissue damage surrounding treated vessels were readily appreciated. The pale, eosinophilic central zone extends a considerable distance into the tissues. This transitions into an inflammatory zone with numerous lymphocytes and a surrounding rim of hemorrhage in the tissues (Fig 6).

### Laboratory values

There were minor variations in laboratory values that in some instances reached statistical significance, but all of the variations remained within normal limits (Table 1). No significant changes were noted in ALT, ALP, total bilirubin, or creatinine. In the experimental group, there was a slight but statistically significant increase in AST and LDH levels 24 h post experiment (*P* = 0.043, *P* = 0.009 respectively).

**Table 1:** Liver function laboratory results.

	Control			Experimental		
	*Pre*	*Post*	*24Hour Post*	*Pre*	*Post*	*24Hour Post*
*AST* *(14–56 U/L)*	*20*.*00±7*.*55*	*16*.*33±3*.*79*	*32*.*33±19*.*09*	*13*.*40±2*.*97*	*13*.*00±2*.*24*	*81*.*20±53*.*45*
*ALT* *(5–78 U/L)*	*31*.*00±3*.*61*	*31*.*00±4*.*58*	*38*.*67±8*.*33*	*25*.*60±2*.*70*	*25*.*00±3*.*16*	*35*.*60±3*.*78*
*ALP* *(164 ±12 U/L)*	*138*.*33±10*.*21*	*145*.*00±9*.*54*	*146*.*00±6*.*24*	*138*.*00±24*.*82*	*144*.*80±26*.*99*	*155*.*40±24*.*89*
*LDH* *(140–1155 U/L))*	*551*.*67±176*.*91*	*479*.*67±77*.*36*	*602*.*33±180*.*72*	*430*.*60±51*.*61*	*416*.*60±52*.*19*	*779*.*00±292*.*41*
*Albumin* *(0*.*5–4*.*3 g/dL)*	*2*.*90±0*.*28*	*2*.*82±0*.*27*	*3*.*21±0*.*27*	*2*.*89±0*.*32*	*2*.*80±0*.*37*	*3*.*12±0*.*34*
*Total Protein* *(2*.*25–8*.*15 g/dL)*	*5*.*42±0*.*87*	*5*.*35±0*.*84*	*6*.*05±0*.*84*	*5*.*35±0*.*66*	*5*.*25±0*.*69*	*5*.*94±0*.*67*

All animals had a mild elevation of sodium, chloride, blood glucose levels, total protein, and albumin levels 24 h post experiment (Table 2). The increase in the total protein and albumin levels along with the higher levels of Na, Cl and BUN 24 hours post experiment may reflect mild volume depletion or dehydration. A mild decrease in potassium was also observed.

**Table 2:** Electrolyte panel and hematology values.

	Control			Experimental		
	*Pre*	*Post*	*24Hour Post*	*Pre*	*Post*	*24Hour Post*
*Na* ***133–153 mEq/L***	*136*.*07±2*.*67*	*135*.*27±1*.*67*	*141*.*60±1*.*65*	*136*.*48±1*.*73*	*134*.*44±0*.*59*	*141*.*26±1*.*66*
*K* ***3*.*1–6*.*2 mEq/L***	*4*.*32±0*.*32*	*4*.*59±0*.*24*	*3*.*59±0*.*07*	*4*.*34±0*.*37*	*4*.*54±0*.*32*	*3*.*91±0*.*37*
*Cl* ***96–117 mEq/L***	*100*.*47±0*.*85*	*99*.*40±0*.*61*	*103*.*03±1*.*55*	*98*.*94±1*.*25*	*97*.*02±1*.*61*	*101*.*16±0*.*88*
*BUN* ***8–24 mg/dL***	*7*.*33±0*.*51*	*7*.*03±0*.*48*	*8*.*43±0*.*85*	*7*.*88±1*.*12*	*7*.*62±1*.*08*	*10*.*86±3*.*38*
*Cr* ***1*.*2–2*.*0 mg/dL***	*0*.*91±0*.*08*	*1*.*05±0*.*07*	*0*.*87±0*.*08*	*1*.*07±0*.*13*	*1*.*14±0*.*13*	*1*.*06±0*.*30*
*Gluc* ***48–135 mg/dL***	*52*.*00±7*.*00*	*64*.*67±16*.*17*	*121*.*00±25*.*16*	*73*.*00±8*.*86*	*89*.*40±14*.*52*	*110*.*80±24*.*86*
*WBC* ***6*.*3–21*.*1*** 1000/μL	*14*.*02±3*.*21*	*14*.*44±0*.*71*	*15*.*87±1*.*27*	*14*.*36±1*.*90*	*14*.*54±2*.*06*	*19*.*56±4*.*27*
*Hgb* ***9–16*.*2*** g/dL	*10*.*27±0*.*49*	*10*.*17±0*.*59*	*11*.*03±0*.*84*	*9*.*30±0*.*71*	*8*.*92±0*.*43*	*10*.*10±0*.*98*

No statistically significant changes in vital signs (heart rate, respiration, or oxygenation) was observed associated with the procedure. All animals received a single dose of buprenorphine at the conclusion of the procedure to provide analgesia in the immediate recovery period with no further analgesia needed in either group in the ensuing 24 hours prior to euthanasia.

## Discussion

### Image-guided chemistry

**Fig 7:**
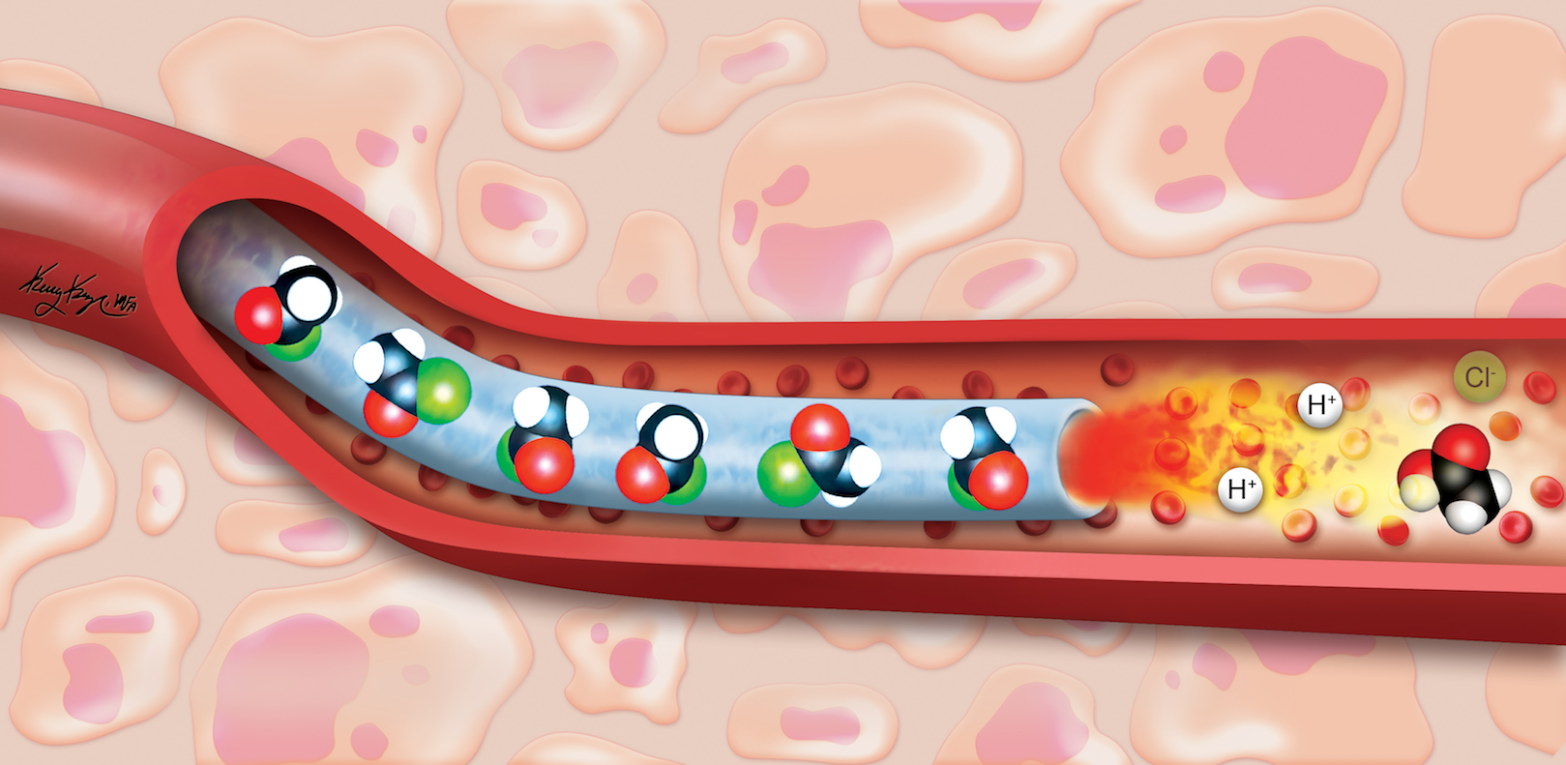
Conceptual view of thermoembolization. As the embolic solution of acid chloride in ethiodized oil exits the catheter, it encounters water in the blood and begins a highly exothermic reaction in situ. The products of the reaction are the incipient carboxylic acid and an equivalent of hydrochloric acid as well.

Prior to beginning investigations in thermoembolization, it was not certain that the desired reaction would indeed occur. It was theoretically possible that the reagent would so strongly partition in the ethiodized oil used as the solvent that it would never encounter a suitable reaction partner. This turned out not to be the case but it is not yet known exactly which compound or compounds the acid chloride reacts with. We consider that the most abundant and therefore the most likely reactive partner functioning as the nucleophile would be the oxygen atom from water (Fig 7). That said, it is also possible that in some cases reaction could occur with other components of either proteins or glycoproteins whether in the blood or as part of either cell membranes or extracellular matrix. Regardless, acid is released, an exotherm occurs, and the results are unambiguous. It is not yet clear what fraction of the observed damage to each aspect according to the mechanisms. These include local acid release (including both HCl and dichloroacetic acid in situ), the temperature increase, the ischemic effects, and likely the interaction between them. Future work with thermal imaging such as MRI thermometry (MRT) may help illuminate this issue to some extent by providing information regarding the thermal dose (peak temperature, duration of exposure). The challenge will lie in the small heated volume relative to a large surrounding thermal mass at body temperature and in using a motion-sensitive method such as MRT in relatively small volumes.

By producing DCA in situ, thermoembolization may make it possible to deliver a high local dose of the drug with beneficial anti-tumor effects. With coagulation of tissues and blockade of vessels preventing rapid washout, the delivered DCA drug could potentially serve as a diffusion reservoir outward in the target region. This would enable a pharmacologic effect at the periphery in the transition zone between dead cells and surviving cells. This notion currently is speculative, however, and will need to be validated. The importance of this rests on the observation that many cancers coopt the aerobic glycolytic pathway. This is known as the Warburg effect and was reported almost a century ago. The topic has seen a resurgence of interest recently [40, 41] as this metabolic program results in the generation of NADPH and enables an increase in biomass, both of which help cancer cells to survive and tumors to grow. DCA blocks pyruvate dehydrogenase kinase (primarily isoform 2), an enzyme which inhibits pyruvate dehydrogenase (PDH). Thus, with PDH activity restored, the drug restores oxidative phosphorylation in the mitochondria and normalizes metabolism. The result is tumor cell death.

In terms of which system to use for the control arm embolization, we chose to perform the procedure with ethiodized oil only, rather than as an emulsion with aqueous doxorubicin. This was done in light of the short time course for the animals after treatment and keeping in mind that doxorubicin is most effective on actively dividing cells that are not present in the healthy pig model system we employed. Use of ethiodized oil also meant that the material would be somewhat more viscous than an emulsion as commonly used in TACE. While this made have resulted in less distal penetration and a relatively proximal distribution, it was still markedly different compared to the experimental group.

### Systemic effects

The lab values from Tables 1 and 2 show minimal evidence of systemic effects from the procedures. In general, liver enzyme levels nor other tests revealed any acute decompensation, suggesting that the procedure was well-tolerated. However, it must be remembered that some values, such as bilirubin, albumen, and total protein could take longer to manifest changes. Fortunately, the observed variances were within the normal range of laboratory values for the duration of the experimen with one exception. We observed a slight change in AST just above baseline levels at 24 h in the experimental group. This may be due to the procedure but the difference was very minor. Longer-term and larger studies may help clarify this issue. The post-embolization syndrome is a well-documented response to treatment, and much larger changes in AST and ALT are typically seen in clinical cases. In addition,frequently there is an elevation in the white blood cell count. In our study, we did not see any evidence of this. Given the small amount of material delivered, this is perhaps not surprising. Further work may uncover such a response with a larger dose. The small dose used was also unlikely, in the event of any systemic exposure, to cause an alteration in pH of the blood or alter the pulmonary venous pressure.

### Imaging

At fluoroscopy, a remarkably short time was necessary to achieve stasis in the experimental group compared to the ethiodized oil. The 24-hour time period prior to euthanasia allowed time for two things to occur. One was for ethiodized oil to travel as distal as possible. This was already clearly occurring even during angiography, and was manifest on CT imaging as a somewhat cloudy appearance of the material in the liver. The second was that this time window allowed the possibility that treated vessels in the experimental group might re-open once the initial response to treatment had subsided. This would enable the embolic material to migrate more distally similar to ethiodized oil and presumably would result in a similar final appearance. However, this was never identified in any case. CT imaging of all animals in the experimental group consistently showed a pattern of attenuation clearly situated in the more proximal subsegmental and segmental vessels. This indicated that over the time period of the study, little or no forward migration of the embolic solution had occurred since the procedure and embolization may well be permanent. Lung CT images showed no evidence of distal embolization in the pulmonary vasculature in any of the animals from either group (data not shown).

### Histology

Conventional staining with hematoxylin and eosin revealed a startling discovery. Based on prior experience and knowledge of reactivity, we predicted some degree of damage in the first or second layer of cells deep to the lumen, perhaps 15–30 microns deep. To our surprise, the area of concentric coagulation necrosis radiating outwardly from the vessel lumen treated vessels was quite large. The distance was in our view remarkable as it extended for several hundred microns away from the vessel wall. As the ethiodized oil control samples appeared identical to untreated liver (data not shown) this is an early glimpse of the potential of the new method. Since the extent of damage could easily encompass the portal triads, suggesting that a minimally invasive, thermochemical segmentectomy is not out of the realm of possibility.

There were several limitations in this study. Although the reaction is known to be exothermic, no temperature data were obtained. The primary goal of this pilot study was to assess initial performance of the technique in vivo, identify practical and logistical issues, and assess for any systemic effects. Given the extremely small dose and the large thermal mass of the liver relative to the dose, it would be unlikely that the energy released would provide a detectable thermal signature. In order to monitor in this fashion, it would be necessary to place a thermocouple immediately adjacent to a segmental artery about to be treated. The utility of this may be questioned as it would achieve only a single point measurement. Alternatively, one could attempt the procedure in a magnet to enable magnetic resonance thermometry. Considering the small size of the vessels and sensitivity of thermometry, motion may preclude this possibility. Another issue is the use of normal tissue rather than a tumor model. However, it is first necessary to establish feasibility in a less complex system. This was an acute study, meaning we could not collect data on how lesions evolve over time. Finally, we did not obtain plasma DCA levels. This would potentially provide a more sensitive estimate of systemic exposure.

## Conclusion

The new concept appears to hold promise while at the same time raising new opportunities for research. There are many questions from a technical standpoint for how to achieve maximal distribution. We do not yet know what concentration is sufficient, how treated areas evolve over time, nor how the technique will perform in tumors. Pharmacokinetic and pharmacodynamics issues will be important to understand. These include getting a better grasp of systemic exposure, determining the distance of diffusion of drug into the treated tissues and the time course for the desired effect. Quantifying the degree to which the drug alters metabolism in the surviving cells in the periphery is another area of interest, as will the immune response. As noted earlier, temperature measurements will help in understanding the thermal dose and how it relates to local acidity. It may also be possible to map the pH in and around the treated area to unravel the role that acidity could play in the interaction with hyperthermia and ischemia. Other applications beyond tumor therapy may become apparent over time. With further refinement, it should be possible to execute a minimally invasive, truly multiplexed approach in a single procedure.
